# Mortality in octogenarian patients with transthyretin amyloidosis treated with tafamidis: a systematic review and meta-analysis

**DOI:** 10.1186/s13023-026-04422-2

**Published:** 2026-06-09

**Authors:** Christiane Santo, Vinicius Machado Correia, Maria L. R. Defante, Bruno Ferlin Saccomani Barbosa, Andre Dabarian, Bruno Vaz Kerges Bueno, Vagner Madrini Junior, Fabio Fernandes

**Affiliations:** 1 Instituto do Coração do Hospital das Clínicas da Faculdade de Medicina da Universidade de São Paulo, Aguiar avenue, 44, Cerqueira César, São Paulo, SP 05403-000 Brazil; 2 Sírio-Libanês Ensino e Pesquisa, São Paulo, SP Brazil; 3 Centro Universitário Redentor, Itaperuna, RJ Brazil; 4 Pontifical Catholic University of Paraná (PUC-PR), Curitiba, Paraná Brazil; 5 Hospital Israelita Albert Einstein, São Paulo, SP Brazil; 6 Myocardiopahies Department, Dr. Enéas de Carvalho Aguiar avenue, 44, Cerqueira César, São Paulo, SP 05403-000 Brazil

**Keywords:** Tafamidis, Transthyretin amyloidosis, ATTR-CM, Octogenarians, Mortality

## Abstract

**Background:**

Transthyretin amyloid cardiomyopathy (ATTR-CM) predominantly affects older adults, and tafamidis has proven efficacy in reducing mortality and hospitalizations in this population. However, its clinical benefit in octogenarians remains uncertain due to limited life expectancy, frailty, and other age-related factors. This meta-analysis evaluates the effect of tafamidis in patients aged ≥ 80 years, aiming to address the gap in evidence regarding the therapy’s impact in this population.

**Methods:**

A systematic review and meta-analysis were conducted by searching PubMed, Embase, and Cochrane databases for studies comparing tafamidis versus control in patients aged ≥ 80 years with transthyretin amyloidosis. The primary outcome was all-cause mortality. Hazard ratios (HRs) were pooled using a random-effects model, and heterogeneity was assessed using I² statistics.

**Results:**

Three studies with a total of 1,866 participants were included (1 randomized controlled trial, 1 prospective cohort, and 1 retrospective study). The majority (77.3%) of patients received tafamidis, and the mean age was 84 years. Tafamidis was associated with a significant reduction in all-cause mortality (HR: 0.51; 95% CI 0.40–0.67; *p* < 0.01; I² = 10.3%). Sensitivity analyses confirmed the robustness of this finding. The tafamidis group showed no increased risk of adverse events compared to controls.

**Conclusion:**

Tafamidis significantly reduces all-cause mortality in octogenarian patients with ATTR-CM, supporting its use as a cornerstone therapy in this population. Further randomized controlled trials are needed to validate these findings and optimize treatment strategies in very elderly patients with advanced disease or comorbidities.

**Supplementary Information:**

The online version contains supplementary material available at 10.1186/s13023-026-04422-2.

## Introduction

Transthyretin amyloidosis cardiomyopathy (ATTR-CM), particularly the wild-type form (ATTRwt), predominantly affects older adults, with most diagnoses occurring in individuals older than 70 years. Some studies estimates a 13.9% prevalence of positive cardiac uptake on bone scintigraphy in men aged ≥ 85 years and a 25% prevalence of systemic amyloidosis in autopsy-based analyses in individuals over 85 [1, 2]. From a clinical perspective, ATTR-CM is associated with heart failure symptoms, reduced functional status, and a high mortality rate, with untreated wild-type ATTR-CM displaying a median survival of approximately 3.6 years [3]. 

In contrast, the variant form of the disease (ATTRv), caused by specific mutations in the TTR gene, exhibits a highly variable prognosis depending on the pathogenic variant. Certain genetic variants are associated with more aggressive cardiac or neurological involvement, which can significantly shorten survival and increase disease burden [4]. 

Tafamidis, the first disease-modifying therapy for ATTR-CM, has demonstrated significant benefits, including improved survival, a reduction in heart failure-related hospitalizations, and preservation of quality of life, as shown in the ATTRAct trial [5]. However, in octogenarian patients, the effectiveness of tafamidis remains unclear due to factors such as limited life expectancy, frailty, and a high prevalence of comorbidities, which pose particular challenges for this population.

To address these knowledge gaps, this meta-analysis was conducted to evaluate the impact of tafamidis on all-cause mortality specifically in octogenarian patients with ATTR-CM. By focusing on this vulnerable and underrepresented subgroup, our study aims to provide evidence to inform clinical practice and guide therapeutic decision-making for this understudied population.

## Methods

The protocol of this systematic review and meta-analysis was registered in the International Prospective Register of Systematic Reviews (PROSPERO) under the registration CRD420251146576. This study was performed in accordance with the recommendations of the Cochrane Collaboration Handbook for Systematic Reviews of Interventions and the Preferred Reporting Items for Systematic Reviews and Meta-Analysis (PRISMA) [6, 7].

### Eligibility criteria and outcomes

The outcome of interest was the all-cause mortality. Eligibility for inclusion in this meta-analysis was based on the following criteria: (1) study population comprising patients aged ≥ 80 years with transthyretin amyloidosis; and (2) comparison between Tafamidis and control groups. The exclusion criteria were: (1) conference abstracts without full-text availability; (2) case reports and case series. No restrictions were applied based on population size, publication date or language.

### Search strategy and data extraction

A comprehensive systematic search of PubMed, Embase, and the Cochrane Library was performed in August 2025, with the complete search strategy detailed in Supplementary material. Two reviewers (S.C. and C. V.) independently performed study selection and data extraction. Any disagreements during the screening or data extraction processes were resolved through discussion and consensus with a third reviewer (D.M.).

### Quality assessment

The risk of bias and applicability concerns were independently assessed by two reviewers (B.B. and S.C) using the ROBINS-I tool. Risk-of-bias plots were generated using the Robvis visualization tool and are presented in the Supplementary material [8]. A funnel plot could not be reported due to the limited number of studies included in the outcome (< 10), in line with Cochrane recommendations [7]. 

### Statistical analysis

Pooled hazard ratios (HRs) with 95% confidence intervals (CIs) were estimated with statistical significance set at *p* < 0.05. All analyses were conducted using the Restricted Maximum Likelihood Estimation random-effects model to account for potential variability across studies. Heterogeneity across studies was assessed using the I² statistics. Leave-one-out sensitivity analyses were conducted to assess the influence of individual studies on the overall pooled estimates. All statistical analyses were conducted using R software (version 4.2.2; R Foundation for Statistical Computing, Vienna, Austria).

## Results

### Study selection

**Fig. 1:**
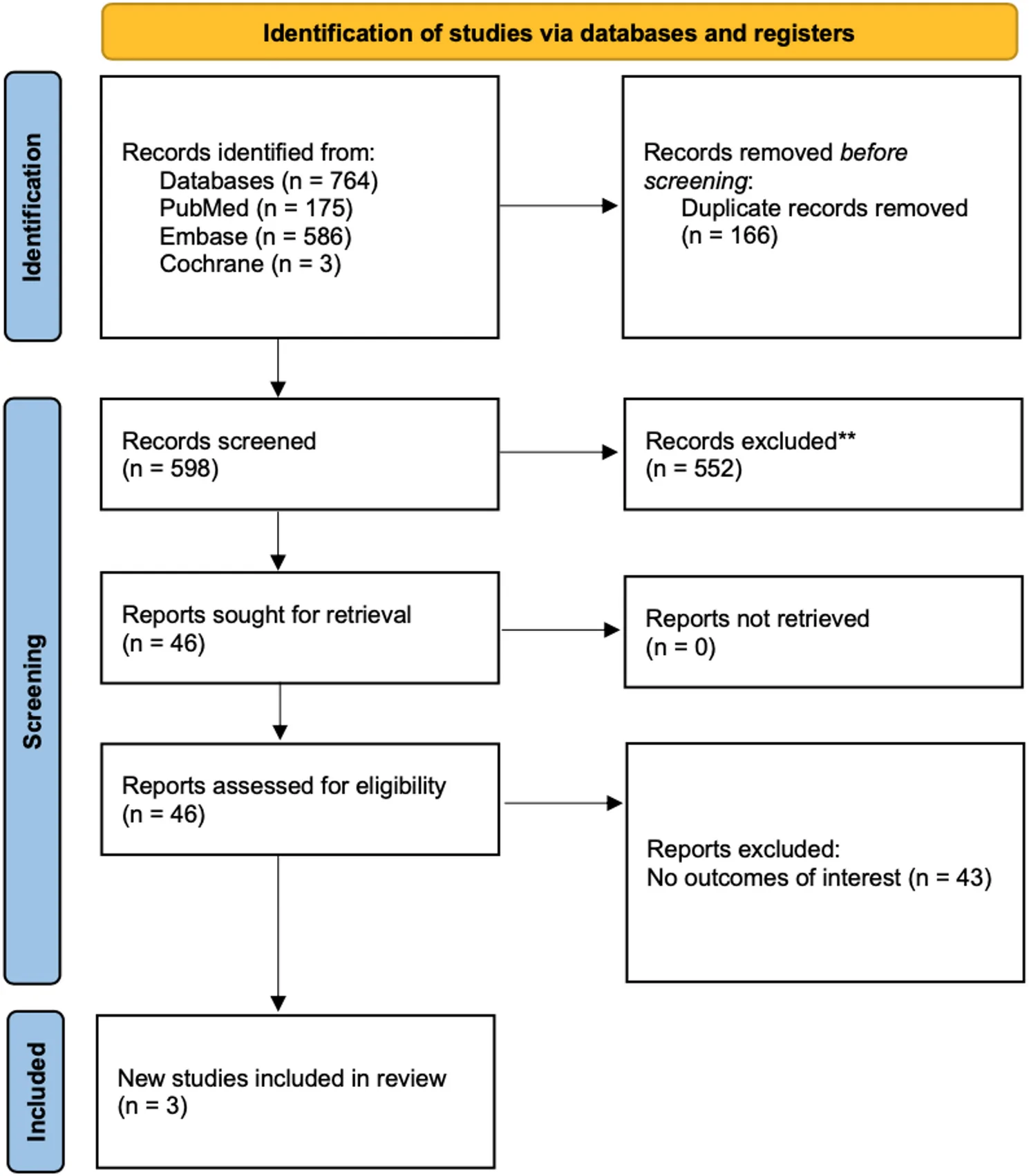
PRISMA flow diagram of study selection

A total of 764 records were identified through database searches (Cochrane, *n* = 3; Embase, *n* = 586; PubMed, *n* = 175). After removal of 166 duplicates, 552 records underwent title and abstract screening. The remaining 46 studies were assessed for eligibility. Ultimately, three studies were included in the systematic review. Figure 1 summarizes the study selection process.

### Study characteristics

Three eligible studies, comprising a total of 1,866 participants, were included in the present meta-analysis. Of these, 1,444 patients (77.3%) received Tafamidis, whereas 422 (22.6%) were assigned to the control group. The pooled mean age across studies was 84.04 years, and the majority of participants were male (78.8%). Most patients were diagnosed with wild-type transthyretin amyloidosis (83.9%). A detailed summary of study-level characteristics is provided in Table 1.

**Table 1:** Characteristics of studies included in the meta-analysis

Study	Tafamidis/Control (n)	NYHA Class III, n (%)	Age, Years*	Male, n (%)	ATTRwt, n (%)	NT-proBNP, pg/mL ^†^	IVS thickness, mm*	LVEF, %*	eGFR, mL/min/1.73 m^2^*
Debonnaire et al. 2025	199/199	60 (30.2)	Tafamidis: 81±6Control: 81±7	304 (76.3)	398 (100)	Tafamidis:3407 (1420-6346)Control:3389 (1322-7038)	Tafamidis: 17±4Control: 16±4	Tafamidis: 55±12Control:54±12	Tafamidis:56±16Control:56±18
Garcia-Pavia et al. 2024	51/37	37	Tafamidis: 83±2.2Control: 82.4±2.6	78 (82.5)	73 (82.5)	3828(2329-5305)	NA	NA	NA
Jobbe-Duval et al. 2025	1194/186	186	85	1075 (77.8)	957 (69.4)	2492.5 (1264-4741.5)	16.4±3.27	53.9±10.8	44.2±14.8

N, number; NYHA; New York Heart Association; ATTRwt, Wild-Type Transthyretin Amyloidosis; NT-proBNP, N-Terminal pro-B-type Natriuretic Peptide; IVS, Interventricular Septal; mm, milimetres; LVEF, Left Ventricular Ejection Fraction; eGFR, estimated Glomerular Filtration Rate

*Mean ± standard deviation

^†^Median (interquartile range)

### Quality assessment

The included studies were judged to present a moderate and serious overall risk of bias. Domain-specific assessments are provided in supplementary material.

### Primary outcome and sensitive analysis

**Fig. 2:**
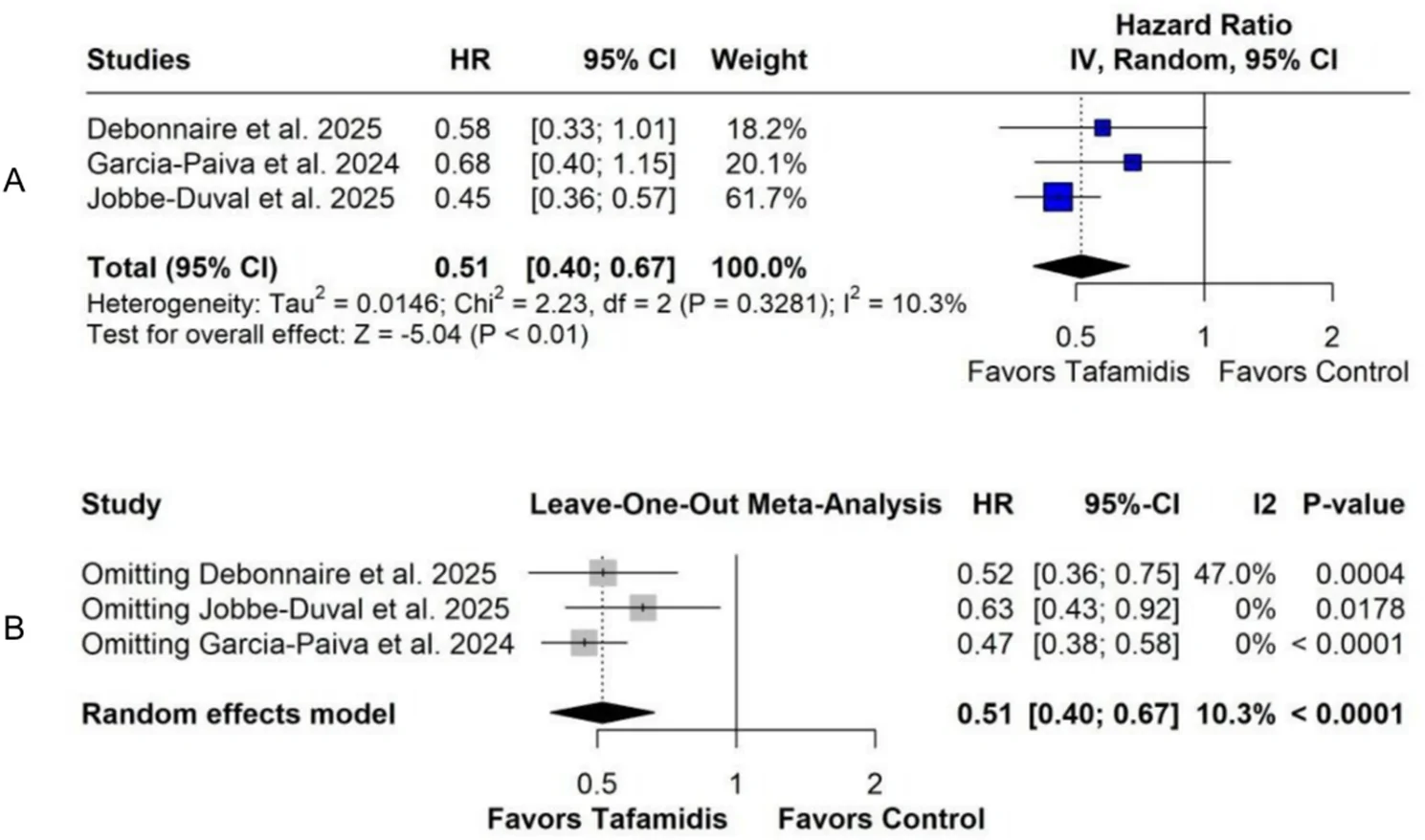
Tafamidis was associated with a reduced risk of all-cause mortality (**A**) and leave-one-out analysis of all-cause mortality (**B**)

The pooled HR for Tafamidis was not associated with an increased risk of all-cause mortality when compared with the control group (HR 0.51; 95% CI 0.40 to 0.67; I²= 10.3%; *p* < 0.01; Fig. 2A). Among the included studies, Jobbe-Duval et al. contributed the greatest weight to the pooled analysis (61.7%), reflecting its larger sample size, whereas Debonnaire et al. and García-Pavía et al. contributed 18.2% and 20.1%, respectively. The leave-one-out sensitivity analysis indicates that the overall effect is robust and not driven by a single study, since the pooled HR remained in favor of Tafamidis and the 95% CI did not cross the line of no effect after omitting the studies individually (Fig. 2B).

## Discussion

In octogenarian patients with ATTR-CM, tafamidis was associated with a significant 49% reduction in all-cause mortality compared to controls, with low heterogeneity across studies. These findings emphasize the clinical relevance of tafamidis as a disease-modifying therapy in this highly vulnerable and underrepresented population, marking the first meta-analysis focused on treatment outcomes in such an elderly subgroup. Importantly, the robustness of these results was confirmed through sensitivity analyses, and no significant safety concerns associated with tafamidis were observed.

The significant mortality reduction observed aligns with robust evidence from prior studies in the general ATTR-CM population, such as the ATTR-ACT trial [5]. This effect is likely driven by tafamidis’ ability to stabilize transthyretin tetramers, reducing the formation of amyloid fibrils and preventing further amyloid deposition in the myocardium [9]. By mitigating disease progression, tafamidis not only improves survival but likely also alleviates symptoms of heart failure and reduces the risk of downstream complications such as heart failure hospitalizations, which are key predictors of mortality in ATTR-CM [10]. These mechanisms of benefit are especially critical in an aging population, where wild-type ATTR-CM predominates, and the disease burden tends to be amplified by comorbidities and frailty [11]. 

Analyzing the included studies, Debonnaire et al. [12] demonstrated that tafamidis-treated patients had a mean age of 85 years, with a high burden of comorbidities, including atrial fibrillation (60.6%) and prior heart failure hospitalizations (36.1%). Although tafamidis was generally well tolerated, 38% of patients discontinued therapy, most commonly due to progressive heart failure, underscoring the challenge of treating advanced disease in this age group. While progressive heart failure is a major driver, other factors such as adverse effects—reported in 16.3% of cases—and undefined causes (18.6%) also contributed to treatment discontinuation. These findings highlight the multifactorial nature of treatment interruption in real-world settings. In this context, particularly among octogenarians, tafamidis may be better understood not only as a survival-oriented therapy but also as an intervention with implications for quality of life. Therefore, decisions regarding treatment continuation or discontinuation should be individualized and ideally guided by a comprehensive geriatric assessment. Importantly, subgroup analysis from Debonnaire’s study revealed that increasing age at treatment initiation was independently associated with increased mortality risk, with nonagenarians exhibiting a 1-year mortality of 38% compared to 15% in younger octogenarians. Furthermore, advanced disease stage at baseline (NAC Stage 3) showed a trend toward increased mortality. Interestingly, baseline NYHA functional class did not significantly predict mortality. However, it is important to emphasize that this was an exploratory analysis with a limited number of patients and a short follow-up period.

The study by Dubal Jobe et al. [13] expanded upon previous findings using a larger cohort of 1,194 treated and 186 untreated patients, predominantly with wild-type ATTR (70.4%). Patients in the treated group presented with less severe disease compared to the placebo group, exhibiting lower NT-proBNP levels and a lower prevalence of NYHA class III/IV. Survival benefits were less apparent in more advanced cases, as cardiac biomarkers did not differ significantly between groups; however, ejection fraction remained higher among treated patients. Importantly, in the analysis of variables independently associated with survival, both NYHA class III/IV status and NT-proBNP levels reached statistical significance in favor of the tafamidis-treated group. The use of propensity score matching in this study helped to mitigate baseline imbalances, thereby strengthening the validity of the observed associations. Additionally, a subanalysis by the same authors focusing on age stratification demonstrated that patients aged 80–85 years in the treatment group had a survival rate of 68%, compared to 58% among those treated above 85 years.

In contrast, the randomized controlled trial by García-Pavía et al. [14], although limited by a smaller sample size (51 patients in the tafamidis group vs. 37 in the placebo group), demonstrated that over a 30-month follow-up period, tafamidis slowed the decline in functional capacity (6MWT), attenuated NT-proBNP elevation, and preserved quality of life (KCCQ-OS). However, no statistically significant differences were observed in all-cause mortality or hospitalization rates, likely due to the limited statistical power of the study.

These findings highlight that while tafamidis provides meaningful clinical benefits in older patients with ATTR-CM, particularly by improving quality of life and delaying functional decline, the impact on survival is less clear and may be modulated by age at initiation, disease severity, and comorbid burden. In very elderly patients, late-stage diagnosis, delayed treatment initiation and high rates of discontinuation often narrow the therapeutic window, complicating the practical implementation of tafamidis in this population [11, 15]. To maximize its utility, early diagnosis is critical, especially for wild-type ATTR-CM, where non-invasive techniques such as bone scintigraphy offer accurate and accessible diagnostic options [16]. Integrating geriatric assessments into clinical workflows is essential for tailoring therapy to address frailty and polypharmacy, enabling a patient-centric approach that balances potential benefits with associated risks. Furthermore, shared decision-making with patients and caregivers regarding the benefits, limitations, and realistic expectations of tafamidis can support adherence and improve outcomes, particularly in light of notable discontinuation rates in clinical practice due to advanced disease or adverse effects.

### Limitations

Nonetheless, our meta-analysis has important limitations. While we included data from three high-quality studies, the overall sample size and the number of included trials were limited. This restriction may impede the ability to evaluate long-term outcomes, such as cardiovascular-specific mortality and arrhythmic events, in this very elderly population. In addition, due to the nature of the available data, cardiovascular-specific mortality, rehospitalisation, and arrhythmic events—highly relevant outcomes in this population, particularly given the complexity and multimorbidity of octogenarian patients—were not consistently reported across the included studies, precluding a robust pooled analysis. Additionally, moderate risk of bias was identified in the observational studies by Debonnaire et al. and Dubal Jobe et al., primarily related to confounders such as frailty and polypharmacy, which were not adequately measured or adjusted for in the analyses. These non-cardiovascular factors may contribute substantially to mortality risk, independently of transthyretin amyloid cardiomyopathy, and the use of all-cause mortality as an endpoint may therefore dilute or obscure the true treatment effect of tafamidis on disease-specific outcomes. Importantly, Debonnaire et al. attempted to mitigate this limitation by employing a propensity score–matched population adjusted for age and cardiovascular risk factors, aiming to reduce bias related to the lack of cardiovascular-specific mortality assessment; even so, tafamidis remained associated with a survival benefit in the treated group. Furthermore, in the same study, Cox proportional hazards regression analysis demonstrated that tafamidis use was associated with improved survival independently of both age at diagnosis and age at treatment initiation.

Furthermore, the randomized controlled trial by García-Pavía et al. demonstrated a serious risk of bias due to the planned crossover of placebo patients to tafamidis during the long-term extension phase, likely leading to an underestimation of mortality differences between groups. In an effort to mitigate these limitations, sensitivity analyses were conducted, demonstrating the robustness of the observed mortality benefits. Additionally, the low heterogeneity across studies supports the validity of these findings, despite inherent methodological differences.

Finally, patient-related and protocol-related heterogeneity among the included studies represents another limitation. Differences in baseline disease severity, participant characteristics (e.g., NYHA functional class, NT-proBNP levels, ejection fraction), and follow-up durations contribute to variability in observed outcomes. For instance, patients initiating treatment at NAC Stage 3 or during late-stage disease may experience diminished benefits compared to those treated earlier. Future research should focus on larger, multicenter randomized controlled trials with more rigorous methodologies, aimed at evaluating treatment efficacy across a broader spectrum of disease severity. These trials should also incorporate geriatric-specific assessments and stratified protocols to better tailor recommendations for octogenarians and even nonagenarians with transthyretin amyloidosis.

## Conclusion

This systematic review and meta-analysis supports evidence that tafamidis is associated with a reduction in all-cause mortality among elderly patients with transthyretin amyloid cardiomyopathy. Although the findings are statistically significant, their clinical interpretation should remain cautious given the overall context of the available evidence. The observed benefits appear to vary according to patient characteristics and disease stage, with potentially more limited effects in advanced disease. While the data support a role for tafamidis in the management of this population, further large-scale, well-designed randomized controlled trials are needed to better define the magnitude of benefit, optimize treatment timing, and refine clinical decision-making in this heterogeneous and vulnerable population.

## Supplementary Information

Below is the link to the electronic supplementary material.

Supplementary Material 1: Risk of Bias of the included studies. Assessment of validity of the included studies according to the Cochrane collaboration risk of bias tool (ROBINS-I).

## Data Availability

All data generated or analysed during this study are included in this published article [and its supplementary information files].

## Abbreviations

ATTR- CM: Transthyretin amyloid cardiomyopathy
ATTRwt: Transthyretin amyloidosis, wild type form
ATTRv: Transthyretin amyloidosis, variant form
TTR: Transthyretin
CI: Confidence interval
HR: Hazard ratio
NAC stage: National amyloidosis center staging system
NYHA: New York heart association functional classification
6MWT: Six minute walk test
KCCQ-OS: Kansas city cardiomyopathy questionnaire overall summary score
NT-pro BNP: N-terminal pro-B-type natriuretic peptide

## Declarations

### Prospective register of systematic reviews (PROSPERO)

CRD420251146576
