# Supplementary material for: Mortality in octogenarian patients with transthyretin amyloidosis treated with tafamidis: a systematic review and meta-analysis
Source: Orphanet J Rare Dis. 2026 Jun 9;21:282. doi: 10.1186/s13023-026-04422-2 (PMC13487931; doi:10.1186/s13023-026-04422-2)
Supplement: Supplementary file 1 [file 13023_2026_4422_MOESM1_ESM.docx]

Risk of Bias of the included studies.

Search strategy Pubmed:

("tafamidis") AND ("octogenarians" OR "wild type"OR "elderly" OR "nonagenarians")

Search strategy Embase:

('tafamidis') AND ('octogenarians' OR 'wild type' OR 'elderly' OR 'nonagenarians')
